# Characteristics of tuberculosis patients in the integrated tuberculosis control model in Chongqing, China: a retrospective study

**DOI:** 10.1186/s12879-020-05304-z

**Published:** 2020-08-05

**Authors:** Liwen Zhang, Wei Xing, Jiani Zhou, Rui Zhang, Yong Cheng, Jin Li, Geng Wang, Shili Liu, Ying Li

**Affiliations:** 1 Department of Social Medicine and Health Service Management, Army Medical University (Third Military Medical University), No.30 Gaotanyan Road, Shapingba District, Chongqing, 400038 China; 2 Chongqing Public Health Medical Center (CPHMC), Chongqing, China

**Keywords:** Tuberculosis, Drug-resistant tuberculosis, HIV-positive tuberculosis, Retreatment

## Abstract

**Background:**

China ranks second in the world in terms of numbers of tuberculosis (TB) cases and is one of the top three countries with the largest number of multidrug-resistant and rifampicin-resistant TB (MDR/RR-TB). It also has high mortality and low cure rates of human immunodeficiency virus (HIV)-positive TB patients. This study aimed to analyse, under the integrated TB control model, the characteristics of TB patients seeking healthcare in the largest designated TB hospital in Chongqing.

**Methods:**

This was a retrospective study of TB registers in a health facility. Record data of 1827 TB patients who had attended the Chongqing Public Health Medical Center (CPHMC) from 1 January to 31 December 2018 were included. The Statistical Package for Social Science (SPSS 18.0; IBM Corporation, Armonk, NY, USA) was used to analyse the data. Counting data were compared using the chi-square test or Fisher’ s exact test. Among the results of the univariate analysis, the variables with statistical significance were included in the binomial stepwise logistic regression, with odds ratio and 95% confidence interval calculated. A two-tailed probability level of *P* < 0.05 was considered statistically significant.

**Results:**

The majority of registered patients were men (1197), of Han ethnicity (1670), aged 21–60 years (1331), farmer/unemployed (1075), and living in county/district (1207). Approximately 24.9% of patients (455/1827) contracted DR-TB, 6% (110/1827) were co-infected with HIV, and 41.0% (749/1827) had drug-related hepatotoxicity. Among those patients, DR-TB was more likely to develop among farmers who received retreatment and had drug-related hepatotoxicity (*P* < 0.05). Women who received retreatment and lived in county/district were less likely to be HIV positive (*P* < 0.05). Compared with farmers, patients who were unemployed were more likely to be HIV positive, and those aged 21–60 years had a higher risk of being tested as HIV positive (*P* < 0.05).

**Conclusion:**

Farmers who received retreatment and had drug-related hepatotoxicity are more susceptible to DR-TB; young unemployed men have a higher risk of contracting HIV-positive TB. The demographic and clinical characteristics of TB patients should be taken into consideration in DR-TB and HIV-positive TB screening in the future.

## Background

Tuberculosis (TB) is one of the top ten causes of death worldwide and is the leading cause of infectious diseases [1]. According to the World Health Organization (WHO)‘s Global TB Report 2019, there were 10.0 (9.0–11,1) million new cases of TB, and 3.4% of new cases and 18% of previously treated cases developed multidrug-resistant TB (MDR-TB) or rifampicin (RFP)-resistant TB (RR/TB) in 2018 [1]. Drug-resistant TB (DR-TB) and TB/human immunodeficiency virus (HIV) co-infection are the challenges in controlling TB (endeavours) worldwide. TB killed 251,000 HIV-positive people globally in 2018, although the number of TB deaths among HIV-positive people has fallen from 620,000 in 2000 to 251,000 in 2018, a decrease of 60, and 8.6% of TB patients are living with HIV [1]. The latest treatment success rates were 85% for drug-sensitive TB, 75% for HIV-associated TB, and 56% for MDR/RR-TB [1]. In many situations, patients not only had TB/DR-TB, but also HIV and other comorbid conditions and complications, which made co-treatment for TB/DR-TB and other diseases more complicated and challenging [2]. It was previously proven that HIV-positive patients are vulnerable to DR-TB, and DR-TB is often associated with higher mortality rates in patients living with HIV [3, 4]. A higher mortality rate was observed in HIV-positive TB patients [5], and TB patients co-infected with HIV were more likely to develop extra-pulmonary disease [6, 7]. Furthermore, hepatotoxicity is one of the most common TB complications caused by first-line drugs (isoniazid [INH], RFP, and pyrazinamide), as these drugs are highly associated with hepatotoxicity [4].

China is burdened with the second largest number of TB and MDR-TB cases with an estimated 886,000 TB patients (including TB/HIV co-infection), 66,000 MDR/RR-TB patients, and 2400 TB/HIV co-infection patients in 2018 [1]. According to the report of the fifth national TB epidemic research in 2010, Chinese TB prevalence is much higher in the western region compared with that in the eastern and central regions with an active TB rate of 695 per 100,000 persons, while those in the eastern and central regions are 291 per 100,000 and 463 per 100,000 [8].

The Chongqing municipality is one of four municipalities directly under the control of the Central Government of China, which is located in southwest China [8]. With over 30 million residents, Chongqing has a high proportion of migrant workers and the highest TB prevalence compared with the other three municipalities [9, 10]. Recently, Chongqing has begun to pay more attention to TB control and has implemented a national TB control programme with great progress [11]. However, the fifth national TB epidemic survey in 2010 indicates that Chongqing’s active TB prevalence rate (> 15 years) and average reported prevalence were both higher than the national average rates [12–14]. The national survey in 2015 indicated that the incidence of TB in Chongqing was 75/100,000, ranking tenth among the top 10 in China [15]. Therefore, TB control in Chongqing faced many challenges, such as the prevalence of MDR-TB and TB/HIV co-infection. Since the implementation of the 12th 5 Year Plan of the National TB Program in 2011, China’s TB control model has transformed from the “Center for Disease Control and Prevention (CDC) model” to the “integrated model” in most regions [16]. In the integrated model, the CDC is responsible for the planning, supervision, and health education of TB control. Designated hospitals are responsible for TB diagnosis and treatment. Primary healthcare facilities are responsible for case management and patient referrals [16]. In the integrated model, TB was diagnosed according to the guidelines of TB control issued in 2017 [17], and the treatment of TB referred to the Guidelines for implementing the national TB control programme in China (2008) [18]. Chongqing has also implemented the integrated model so that TB patients can receive therapy in designated hospitals since 2016. To better understand the progress made in Chongqing’s TB control as part of the integrated model, it is essential to reanalyse TB epidemiology in Chongqing. This study aimed to understand the characteristics of TB epidemiology in Chongqing, particularly among DR-TB and TB/HIV co-infection patients. It also aimed to identify the population at higher risk of DR-TB and HIV-positive TB to provide evidence that can serve as a basis for making responsive strategies to end the spread of TB in Chongqing.

## Methods

### Setting and participants

We performed a retrospective study using the registered data collected. The Chongqing Public Health Medical Center (CPHMC) is the biggest tertiary hospital with Grade A level of prevention and treatment for infectious diseases in Chongqing. In addition, CPHMC, as the Medical Quality Control Center of TB, HIV, and respiratory infectious diseases, is the officially designated hospital for diagnosis and treatment of TB, MDR-TB, and HIV in Chongqing [19]**.** Therefore, we chose the recorded data from the TB reporting system in CPHMC to represent TB patients in Chongqing, China. The TB reporting system includes the following information: individual data on TB patients; TB diagnosis, treatments, and outcome; quarterly National TB Program activity reports from TB dispensaries at all levels; and annual reports on funding for TB control activities [20]**.** TB cases were diagnosed based on the Chinese clinical guidelines for TB diagnosis and treatment in CPHMC. CPHMC reports all notified TB patients to the China Information System for Disease Control and Prevention (CIS-DCP). A total of 1827 patients with pulmonary TB attended CPHMC to receive TB treatment from 1 January to 31 December 2018 and were included in our study.

### Data sources and statistical analyses

The standard form was used to collect the data of each TB patient during the study period. The record system in CPHMC includes age, gender, occupation, living region, ethnicity, symptoms, and drug-related hepatotoxicity, viral hepatitis, TB type, and HIV infection. Observational variables in this study included demographic and clinical characteristics of the patients and factors associated with DR-TB and TB/HIV co-infection. Several definitions related to TB were used in this study. DR-TB consists of MR-TB, MDR-TB, XDR-TB, PDR-TB, RR-TB, and Hr-TB in our study. Mono-resistant TB (MR-TB) refers to *Mycobacterium tuberculosis* strains with resistance to one first-line anti-TB drug [5]**.** MDR-TB is defined as TB that is simultaneously resistant to the two most powerful anti-TB drugs, RFP and INH, with or without resistance to other first-line drugs and requires treatment with second-line drugs [1]**.** XDR-TB is defined as MDR-TB plus resistance to at least one drug in both fluoroquinolones and second-line injectable agents (amikacin, capreomycin, or kanamycin), which are the two most important classes of medicines in MDR-TB treatment [21]**.** Poly-drug-resistance (PDR-TB) refers to resistance to more than one first-line anti-TB drug, other than isoniazid (INH) and RFP [22]**.** RR-TB is considered susceptible to INH, resistant to INH, or resistant to other first-line TB drugs or second-line TB drugs that also require treatment with a second-line regimen [14]**.** INH-resistant TB (Hr-TB) refers to *M. tuberculosis* strains with resistance to INH and susceptibility to RFP confirmed in vitro [21]**.** The results of this study were reported following the Strengthening the Reporting of Observational studies in Epidemiology (STROBE) statement for reports of observational studies.

All patients’ registration system information were collected from the Chongqing Public Health Medical Center. The Statistical Package for Social Science (SPSS 18.0; IBM Corporation, Armonk, NY, USA) was used to analyse the data. Counting data were compared using the chi-square test or Fisher’ s exact test to explore the differences in demographic and clinical characteristics among DR-TB and HIV-positive TB patients. Based on the results of univariate analysis, the variables with statistical significance were included in the binomial stepwise logistic regression, and odds ratio and its corresponding 95% confidence interval were calculated. A two-tailed probability level of *P* < 0.05 was considered significant.

## Results

### Patients’ characteristics

The demographic and clinical characteristics of patients are illustrated in Table 1. A total of 1827 patients were included in our study, and majority of them (65.5%, *n* = 1197) were men. Among them, 72.9% (*n* = 1331) were aged 21–60 years, 91.4% (*n* = 1670) were of Han ethnicity, and 66.1% (*n* = 1207) were from the county/district of Chongqing. A total of 565 (30.90%) patients were farmers, 510 (27.9%) were unemployed, 257 (14.1%) were currently non-working, and 174(9.5%) were students. Approximately 22.9% (*n* = 418) of the patients had retreatment of TB and 455 (24.90%) had DR-TB. Notably, 87.03% (*n* = 396) DR-TB of the patients had MDR-TB. In addition, 110 (6%) TB patients were co-infected with HIV.

**Table 1:** Demographic and clinical characteristics of TB patients

Characteristics	Categories	Number (N)	Percent (%)
Gender(*N* = 1827)	Male	1197	65.50
	Female	630	34.50
Ethnicity(*N* = 1827)	Han	1670	91.40
	Others	157	8.60
Occupation(*N* = 1826)	Farmer	565	30.90
	Unemployed	510	27.90
	Currently non-working	257	14.10
	Student	174	9.50
	Housekeeping	101	5.50
	Manual worker	68	3.70
	Others	151	8.30
Living region(*N* = 1827)	Central urban	620	33.90
	County/district	1207	66.10
Age(*N* = 1827)	≤20	224	12.30
	21–40	692	37.90
	41–60	639	35.00
	≥61	272	14.90
Symptoms(*N* = 915)	Typical symptoms	464	50.70
	Others	451	49.30
Retreatment(*N* = 1827)	Yes	418	22.90
	No	1409	77.10
Drug-related hepatotoxicity(*N* = 1827)	Yes	749	41.00
	No	1078	59.00
Viral hepatitis(*N* = 1827)	Yes	44	2.40
	No	1783	97.60
AIDS/HIV(*N* = 1827)	Yes	110	6.00
	No	1717	94.00
DR-TB(*N* = 455)	MDR-TB	396	87.03
	MR-TB	18	4.00
	XDR-TB	16	3.50
	PDR-TB	13	2.86
	Hr-TB	2	0.44
	RR-TB	10	2.2

Notes: Missing data were excluded

### Factors associated with DR-TB and TB/HIV co-infection

Results of χ^2^ tests (Table 2) indicated that patients of Han ethnicity were less likely to have DR-TB compared with other ethnicities (23.90% vs. 35.70%, *P* = 0.002). Housekeepers were more likely to have DR-TB than other professions (*P* < 0.001). Patients who lived in suburban counties/districts were more likely to have DR-TB (27.10% vs. 20.60%, *P* = 0.001) than those living in central urban areas. Patients aged between 21 and 60 years had a higher risk of having DR-TB than those aged less than 21 years and more than 60 years (*P* = 0.010). Patients in retreatment (95.20% vs. 4.00%, *P* < 0.001) and with drug-related hepatotoxicity (43.70% vs. 11.90%, *P* < 0.001) were more likely to have DR-TB. However, HIV-positive TB patients were less likely to have DR-TB than HIV-negative TB patients (8.20% vs. 26.00%, *P* < 0.001). No differences were observed in sex, symptoms (excluded revisit patients as they had no initial symptoms based on their clinical records), and viral hepatitis between drug-sensitive TB patients and DR-TB patients (*P* > 0.05).

**Table 2:** Univariate analysis of factors association with DR-TB patients and TB/HIV co-infection

Variables	Categories	DR-TB (*N* = 455)	P(χ^2^test)	TB/HIV co-infection (*N* = 110)	P (χ^2^test)
Gender	Male	292 (64.2)	0.495	87 (7.3)	0.002^*^
	Female	163 (25.90)		23 (3.70)	
Ethnicity	Han	399 (23.90)	0.002^*^	104 (6.20)	0.292
	Others	56 (35.70)		6 (3.80)	
Occupation	Farmer	144 (25.50)	< 0.001^*^	32 (5.70)	< 0.001^*^
	Unemployed	121 (23.70)		56 (11.00)	
	Currently non-working	70 (27.20)		7 (2.70)	
	Student	27 (15.50)		2 (1.10)	
	Housekeeping	44 (43.60)		4 (4.00)	
	Manual worker	16 (23.50)		3 (4.40)	
	Others	33 (21.90)		6 (4.00)	
Living region	Central urban	128 (20.60)	0.001^*^	51 (8.20)	0.007^*^
	County/district	327 (27.10)		59 (4.90)	
Age	≤20	42 (18.700)	0.010^*^	2 (0.90)	0.005^*^
	21–40	186 (26.90)		47 (6.80)	
	41–60	173 (27.10)		46 (7.20)	
	≥61	54 (19.90)		15 (5.50)	
Retreatment	Yes	398 (95.20)	< 0.001^*^	8 (1.90)	< 0.001^*^
	No	57 (4.00)		102 (7.20)	
Drug-related hepatotoxicity	Yes	327 (43.70)	< 0.001^*^	53 (7.10)	0.133
	No	128 (11.90)		57 (5.30)	
Viral hepatitis	Yes	10 (22.70)	0.861	2 (4.50)	1.000
	No	445 (25.00)		108 (6.10)	
AIDS/HIVS	Yes	9 (8.20)	< 0.001^*^		
	No	446 (26.00)			
Symptoms	Typical symptoms	64 (13.80)	0.158		
	Others	48 (10.60)			

Notes: Missing data were excluded.^*^P < 0.05.

As Table 2 shows, male TB patients were more likely to be HIV positive compared with women (7.30% vs. 3.70%, *P* = 0.002). Patients who were unemployed were more likely to be HIV positive (*P* < 0.001) than those who had jobs. Patients living in central urban areas were more likely to be HIV positive than those living in county/district (8.20% vs. 4.90%, *P* = 0.007). Patients aged 21–60 years had a much higher risk of being HIV positive than those aged less than 21 years and more than 60 years (*P* = 0.005). However, patients who received retreatment were less likely to be HIV positive than those who did not receive retreatment (1.90% vs. 7.20%, *P* < 0.001). No differences were observed among different ethnicities, existence of drug-related hepatotoxicity, and viral hepatitis between HIV-negative patients and HIV-positive patients (*P* > 0.05).

Multivariate analysis of factors associated with DR-TB and TB/HIV co-infection is presented in Table 3. The assignment of variables is shown in Additional file 1. The binomial stepwise logistic regression is shown in Table 3. TB type and HIV infectious status were the dependent variables (Y). Gender, ethnicity, occupation, living region, age, retreatment, drug-related hepatotoxicity, and HIV were the independent variables (X). Results indicated that patients who received retreatment and had drug-related hepatotoxicity were more likely to have DR-TB. However, compared with farmers, patients who worked in other occupations were less likely to have DR-TB. The results showed that patients who received retreatment and lived in county/district were less likely to be HIV positive. Female patients were less likely to be HIV/AIDS positive than male patients. However, compared with farmers, patients who were unemployed were more likely to be HIV-positive, and patients aged 21–60 years had a higher risk of being HIV-positive than those aged less than 21 years and more than 60 years.

**Table 3:** Binomial stepwise logistic regression analysis for factors associated with DR-TB and TB/HIV co-infection

Variables	Categories	DR-TB OR(95%CI)	TB/HIV co-infection OR(95%CI)
Gender	Male		Reference
	Female		0.468 (0.286,0.765)
Occupation	Farmer	Reference	Reference
	Others	0.340 (0.128.0.905)	
	Unemployed		2.013 (1.256,3.226)
Living region	Central urban		Reference
	County/district		0.644 (0.429,0.968)
Age	≤20		Reference
	21–40		5.817 (1.225,27.631)
	41–60		5.313 (1.093,25.825)
Retreatment	No	Reference	Reference
	Yes	433.763 (248.261,757.874)	0.232 (0.111,0.484)
Drug-related hepatotoxicity	No	Reference	
	Yes	2.676 (1.683,4.253)	

Notes: Only *P* < 0.05 presented

## Discussion

Our study described the epidemiology of TB patients from CPHMC and evaluated the factors associated with DR-TB and TB/HIV co-infection. Results showed that most TB patients in Chongqing were young and middle aged, which is consistent with the reports in other regions of China [23, 24]. TB cases are more likely to cluster among disadvantaged groups such as the poor, hungry, and ethnic minorities, and most TB patients were farmers, unemployed, and those living in county/district areas [1, 25], which were considered as the populations with relatively poor social economic status, great financial pressure, and non-ideal living conditions [10]. This study consistently found that farmers and unemployed individuals who lived in county/district had higher susceptibility to TB in Chongqing. In 2015, the WHO proposed a core principle, included in the End TB strategy, focusing on social protection in the context of TB, particularly poor and vulnerable populations [26]. Furthermore, the WHO’s 2019 Global TB Report also recommended providing social support for vulnerable people, such as transport, food packages, and cash-transfer schemes to avoid catastrophic costs for TB patients and their households [1]. Therefore, the Chongqing TB prevention and control strategy needs to continue and focus on people with disadvantages in order to address the social determinants of TB.

Students are vulnerable to TB; hence, more TB control and preventive efforts should be made to protect students in schools [4]. TB prevalence among Chinese students (1520/100,000) is much higher than that (459/100,000) among the general population [27]. Chongqing was one of the regions with the highest TB burden among undergraduate students in China [28]. Several TB outbreaks among students have been recently reported in Chongqing [29–31]. This study also observed that almost 10% of the patients were students in this region. Previous studies have shown that students in this region have poor knowledge of TB [32]. TB programmes in this region need to make more efforts to protect students, particularly those living in school. These efforts include health education and behavioural interventions.

The DR and MDR rates in Chongqing have also increased recently. In 2009, the DR and MDR rates in smear-positive TB patients living in urban areas were 27.62 and 10.50%, respectively, and the MDR rates in new TB and retreatment TB patients were 6.99 and 23.68%, respectively [33]. By 2015, the DR and MDR rate in smear-positive TB patients were 35.6 and 23.8%, while the MDR rates of new TB cases and retreatment TB patients were 8.7 and 28.8%, respectively [34]. Approximately 24.90% of TB patients in our study had DR-TB, while 87.03% of DR-TB patients had MDR-TB, which indicates that 21.67% of TB patients had MDR-TB. This study found that the MDR-TB rate was slightly lower than 23.8%, which was the value reported by another study in Chongqing in 2017, and higher than 6.8%, the value reported in the fifth national TB epidemiological survey in 2010 [8, 34], indicating that the prevalence of DR-TB in Chongqing is serious and cannot be ignored.

Since the first HIV infection was reported in Chongqing, the epidemic has developed particularly fast in recent years. The number of cases reached 32,589 in 2015 [35]. The TB/HIV co-infection rate in Chongqing was 6% in 2015, which was much higher than the national average level of 2% [36]. It is more difficult for TB patients living with HIV to receive TB treatment due to the increasing financial burden, overlapping drug toxic effects, and fragmented care between separated TB and HIV healthcare programmes; therefore, TB patients co-infected with HIV are more likely to develop DR-TB and have treatment failure [37, 38]. Some studies pointed out that the risk factors of both DR-TB and HIV could be highly identical, so that many DR-TB patients are co-infected with HIV, which has aggregated the disease burden and increased poor outcomes [39–41]. Therefore, it is essential for healthcare facilities to perform drug-susceptibility tests in every patient with TB and HIV [26].

In addition, 41% of patients in this hospital in Chongqing developed drug-related hepatotoxicity caused by first-line drugs (INH, RFP, and pyrazinamide). This could affect patients’ adherence to treatment and increase the risk of DR-TB. There is an urgent need for more effective anti-TB drugs to shorten the treatment duration, for better healthcare accessibility (both location and capable laboratories), and for faster TB/DR-TB diagnosis to avoid clinical deterioration, death, and transmission [42].

Patients who received retreatment had a higher risk of DR-TB, which was consistent with the results of previous studies [43, 44]. TB patients with drug-related hepatotoxicity were more likely to have DR-TB, similar to previous observations from other studies [45]. Moreover, farmers were at risk for DR-TB. Farmers had poor accessibility to TB-related healthcare as they lived in remote mountainous areas with poor economic status [46], increasing the risk of developing DR-TB.

Previous studies reported that HIV and DR-TB were the risk factors for each other [39–41, 47]. However, this study found no association between HIV-positive and DR-TB patients among TB patients in Chongqing. This result may be explained by the high mortality and default rate reported among TB/HIV co-infection patients [3, 4, 48]. Patients living in the central urban areas of Chongqing had higher risks of acquiring HIV because the prevalence of HIV existed mainly in central urban areas, and the incidence of HIV was high among men who had sex with men living mainly in the central urban areas of Chongqing [49]. Therefore, this might explain why male TB patients had a higher risk of HIV co-infection in our study. Patients aged 21–60 years had higher risks of being HIV positive than those aged less than 21 years and more than 60 years. This may also be attributed to less sexual behaviours and non-intravenous drug use reported by previous studies in recent years [49]. The younger population engaged in more HIV risk activities. In addition, unemployed patients were more likely to have HIV than farmers, as the unemployed population may perform more HIV risk behaviours with more free time and poorer economic conditions. Besides, patients who received retreatment had a lower risk of being HIV positive, which may be due to the high mortality and default rate of HIV-positive TB patients. A low retreatment rate was observed among HIV-positive TB patients.

Our study has some limitations. First, cases registered at other designated hospitals and general hospitals were not included in our data collection, which would result in relatively lesser representative ability of our results. Second, our study population included those who attended CPHMC for treatment from 1 January to 31 December 2018. Therefore, a few treatment data were missed, such as treatment outcomes, undetected HIV co-infected DR-TB cases, and undetected DR-TB cases. These missing data could influence the results of our data analysis, which may have led to bias in the measurement of the association between HIV-positive TB and DR-TB.

## Conclusion

Young men and socially disadvantaged people, including students, were at higher risk of TB. DR-TB and TB/HIV co-infection remain a challenge in TB prevention and control in Chongqing. Hence, healthcare services for TB and HIV must be integrated to facilitate early identification and treatment of TB/HIV-positive co-infection patients among the unemployed, young, and urban area TB patients.

## Supplementary information

**Additional file 1.**

## Data Availability

The datasets generated and/or analysed during the current study are available from the corresponding author on reasonable request.

## Abbreviations

CDC: Center for Disease Control and Prevention
CIS-DCP: China Information System for Disease Control And Prevention
CPHMC: The Chongqing Public Health Medical Center
DR-TB: Drug-Resistant Tuberculosis
Hr-TB: Isoniazid-Resistant Tuberculosis
MDR-TB: Multidrug-Resistant Tuberculosis
MR-TB: Mono-Resistant Tuberculosis
PDR-TB: Poly-Drug-Resistance Tuberculosis
RR/TB: Rifampicin-Resistant Tuberculosis
STROBE: Strengthening the Reporting of Observational studies in Epidemiology
TB: Tuberculosis
WHO: World Health Organization
