# Supplementary material for: Characteristics of tuberculosis patients in the integrated tuberculosis control model in Chongqing, China: a retrospective study
Source: BMC Infect Dis. 2020 Aug 5;20:576. doi: 10.1186/s12879-020-05304-z (PMC7404911; doi:10.1186/s12879-020-05304-z)
Supplement: Supplementary file 1 — Additional file 1. [file 12879_2020_5304_MOESM1_ESM.docx]

## Appendix

| Appendix 1.Binomial stepwise logistic regression assignment of DR-TB and TB / HIV co-infection | | | | |
| --- | --- | --- | --- | --- |
|  | Factor | Variables | | Assignment |
| DR-TB | TB type | Y | | 0=Drug-sensitive TB, 1=DR-TB |
|  | Ethnicity | X1 | | 0=Han, 1=Others |
|  | Occupation | X2 | | 1=Farmer, 2= Unemployed, 3=Currently non-working, 4=Student, 5=Housekeeping, 6=Manual Worker, 7=Others |
|  | Living region | X3 | | 1=Central urban, 2=County/district |
|  | Age | X4 | | 1=≤20, 2=21-40, 3=41-60, 4=≥61 |
|  | Retreatment | X5 | | 0=No, 1=YES |
|  | Drug-related hepatotoxicity | X6 | | 0=No, 1=YES |
|  | AIDS/HIV | X7 | | 0=No, 1=YES |
| TB/HIV co-infection | HIV | Y | | 0=HIV-negative, 1=HIV-positive |
|  | Gender | X1 | | 0=Male, 1=Female |
|  | Occupation | X2 | | 1=Farmer, 2=Unemployed, 3=Currently non-working, 4=Student, 5=Housekeeping, 6=Manual Worker, 7=Others |
|  | Living region | X3 | | 1=Central urban, 2=County/district |
|  | Age | X4 | | 1=≤20, 2=21-40, 3=41-60, 4=≥61 |
|  | Retreatment | X5 | 0=No, 1=YES | |
